# Supplementary material for: Causal Relationship between Aging and Anorexia Nervosa: A White-Matter-Microstructure-Mediated Mendelian Randomization Analysis
Source: Biomedicines. 2024 Aug 16;12(8):1874. doi: 10.3390/biomedicines12081874 (PMC11351342; doi:10.3390/biomedicines12081874)
Supplement: Supplementary file 1 [file biomedicines-12-01874-s001.zip › Supplementary Figure/Figure S4/Singe SNP plot of MR analysis for TL to ubm-b-1491.pdf]

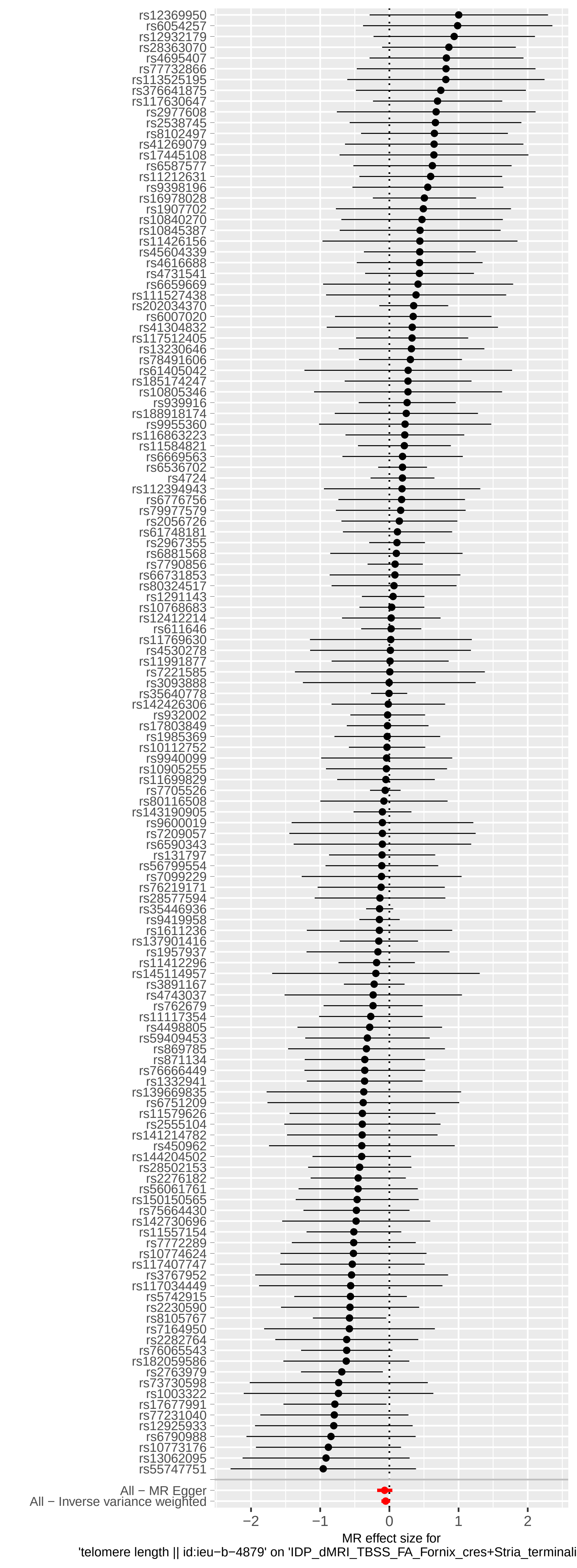
rs12369950
rs6054257
rs12932179
rs28363070
rs4695407
rs77732866
rs113525195
rs376641875
rs117630647
rs2977608
rs2538745
rs8102497
rs41269079
rs17445108
rs6587577
rs11212631
rs9398196
rs16978028
rs1907702
rs10840270
rs10845387
rs11426156
rs45604339
rs4616688
rs4731541
rs6659669
rs111527438
rs202034370
rs6007020
rs41304832
rs117512405
rs13230646
rs78491606
rs61405042
rs185174247
rs10805346
rs939916
rs188918174
rs9955360
rs116863223
rs11584821
rs6669563
rs6536702
rs4724
rs112394943
rs6776756
rs79977579
rs2056726
rs61748181
rs2967355
rs6881568
rs7790856
rs66731853
rs80324517
rs1291143
rs10768683
rs12412214
rs611646
rs11769630
rs4530278
rs11991877
rs7221585
rs3093888
rs35640778
rs142426306
rs932002
rs17803849
rs1985369
rs10112752
rs9940099
rs10905255
rs11699829
rs7705526
rs80116508
rs143190905
rs9600019
rs7209057
rs6590343
rs131797
rs56799554
rs7099229
rs76219171
rs28577594
rs35446936
rs9419958
rs1611236
rs137901416
rs1957937
rs11412296
rs145114957
rs3891167
rs4743037
rs762679
rs11117354
rs4498805
rs59409453
rs869785
rs871134
rs76666449
rs1332941
rs139669835
rs6751209
rs11579626
rs2555104
rs141214782
rs450962
rs144204502
rs28502153
rs2276182
rs56061761
rs150150565
rs75664430
rs142730696
rs11557154
rs7772289
rs10774624
rs117407747
rs3767952
rs117034449
rs5742915
rs2230590
rs8105767
rs7164950
rs2282764
rs76065543
rs182059586
rs2763979
rs73730598
rs1003322
rs17677991
rs77231040
rs12925933
rs6790988
rs10773176
rs13062095
rs55747751
All − MR Egger
All − Inverse variance weighted
−2
−1
0
1
2
MR effect size for
'telomere length || id:ieu−b−4879' on 'IDP_dMRI_TBSS_FA_Fornix_cres+Stria_terminali
